# Modelling and manipulation of aphid-mediated spread of non-persistently transmitted viruses^☆^

**DOI:** 10.1016/j.virusres.2019.197845

**Published:** 2020-02

**Authors:** John P. Carr, Trisna Tungadi, Ruairí Donnelly, Ana Bravo-Cazar, Sun-Ju Rhee, Lewis G. Watt, J. Musembi Mutuku, Francis O. Wamonje, Alex M. Murphy, Warren Arinaitwe, Adrienne E. Pate, Nik J. Cunniffe, Christopher A. Gilligan

**Affiliations:** a Department of Plant Sciences, University of Cambridge, Cambridge CB2 3EA, UK; b Biosciences Eastern and Central Africa-International Livestock Research Institute (BecA-ILRI) Hub, P.O. Box 30709-00100, Nairobi, Kenya; c International Centre of Insect Physiology and Ecology, 30772-00100 Nairobi, Kenya

**Keywords:** Insect vector, Markov chain, Virus acquisition, Epidemiology, Inoculation, Argonaute

## Abstract

Aphids vector many plant viruses in a non-persistent manner i.e., virus particles bind loosely to the insect mouthparts (stylet). This means that acquisition of virus particles from infected plants, and inoculation of uninfected plants by viruliferous aphids, are rapid processes that require only brief probes of the plant’s epidermal cells. Virus infection alters plant biochemistry, which causes changes in emission of volatile organic compounds and altered accumulation of nutrients and defence compounds in host tissues. These virus-induced biochemical changes can influence the migration, settling and feeding behaviours of aphids. Working mainly with cucumber mosaic virus and several potyviruses, a number of research groups have noted that in some plants, virus infection engenders resistance to aphid settling (sometimes accompanied by emission of deceptively attractive volatiles, that can lead to exploratory penetration by aphids without settling). However, in certain other hosts, virus infection renders plants more susceptible to aphid colonisation. It has been suggested that induction of resistance to aphid settling encourages transmission of non-persistently transmitted viruses, while induction of susceptibility to settling retards transmission. However, recent mathematical modelling indicates that both virus-induced effects contribute to epidemic development at different scales. We have also investigated at the molecular level the processes leading to induction, by cucumber mosaic virus, of feeding deterrence versus susceptibility to aphid infestation. Both processes involve complex interactions between specific viral proteins and host factors, resulting in manipulation or suppression of the plant’s immune networks.

## Highlights

• Non-persistent transmission is the most common mode for aphid-mediated virus transmission.
• Many non-persistently transmitted viruses indirectly modify aphid behavior in ways that may enhance transmission.
• Mathematical modeling confirms that enhancing host attractiveness while deterring aphid settling enhances local transmission.
• Modeling predicts that encouraging settling promotes winged aphid production, which may enhance long distance transmission.
• Disrupting host-vector interactions may provide a sustainable means to protect crops.

## 1 Introduction

Arthropods and insects in particular transmit the majority of plant-infecting viruses (Canto et al., 2009; Jones, 2014). The vectors most frequently encountered are hemipteran insects i.e., aphids, whiteflies, planthoppers, leafhoppers and related insects that possess probing mouthparts called stylets (Hull, 2014). Virus transmission by hemipteran insects takes several forms. In persistent (circulative) transmission, viruses are ingested by the vector and circulate within the insect having passed from the gut to the hemocoel. Eventually the virions reach the salivary glands and the insect becomes competent to inoculate plants during feeding. In many cases of persistent transmission of viruses there is no actual replication of the virus in insect cells. Whether or not the virus is capable of replication in insect cells, vectors typically have to ingest the phloem sap of infected plants over a prolonged period since virus acquisition can take hours (Hogenhout et al., 2008; Mauck et al., 2012; Ng and Falk, 2006). For viruses that are transmitted by vectors in a non-persistent or semi-persistent manner, particles are carried in the insect mouthparts for shorter times (non-persistent, minutes to hours and semi-persistent, hours to days). This contrasts with persistently transmitted viruses, which may be carried for the remainder of the vector’s lifespan and in some cases can be passed on the next generation (Hull, 2014). Acquisition of non-persistently or semi-persistently transmitted viruses does not require prolonged feeding from the phloem of infected plants (Hull, 2014). Semi-persistently transmitted virus particles bind to receptors in the insect foregut and virions of non-persistently transmitted viruses bind to receptors located in the common duct of the stylet near the tip of the maxillary stylet in a region called the acrostyle (Liang and Gao, 2017; Webster et al., 2017, 2018).

Because virions of non-persistently transmitted viruses bind very loosely to the acrostyle receptors, these viruses are acquired rapidly during initial probes of an infected plant’s epidermal cells and also are lost rapidly when an aphid ejects saliva through its stylet (Webster et al., 2017, 2018). Work employing the electrical penetration graph method, an electrophysiological technique used to monitor insect feeding activity, showed that acquisition of non-persistently transmitted viruses by aphids requires stylet penetrations of epidermal cells of infected plants that last as little as 3–5 seconds (Moreno et al., 2012; Powell, 2005; Powell et al., 1995). More recent work with a genetically engineered strain of cucumber mosaic virus (CMV) that expresses a fusion between the viral 2b protein and the green fluorescent protein showed that virus replication occurred first in epidermal cells following inoculation by aphid vectors (Krenz et al., 2015). This provided additional evidence that the most efficient means of inoculation for non-persistently transmitted viruses is provided by brief penetrations of plant epidermal cell membranes by the stylet.

The remainder of this article focuses on non-persistent virus transmission. Our objective is to integrate current understanding of viral manipulation of plant vectors in non-persistent virus transmission with the roles of specific viral gene products in manipulating the host and vector, and to consider the epidemiological consequences of viral manipulation of host-vector interactions.

## 2 Viral manipulation of plant–vector interactions

There is compelling evidence that certain genes of plant viruses exert extended phenotypes i.e., these parasite genes influence the expression of host genes in ways that ultimately benefit the virus (Dawkins, 1982). Among the host genes altered in expression by infection are those involved in the biosynthesis of insect-attracting and insect-repelling secondary metabolites and genes involved in defence against insect infestation (see Section 3). The resulting changes in plant biochemistry and defence alter the interactions of infected host plants with vectors and may have profound effects on epidemiological processes that benefit the virus (see Section 4). Although changes in plant biochemistry and defence status that favour transmission were previously assumed to be only incidental effects of virus infection, this assumption has become less tenable as more evidence accumulates of virus-host-vector co-evolution (Chesnais et al., 2019; Groen et al., 2017; Mauck et al., 2019).

**Fig. 1:**
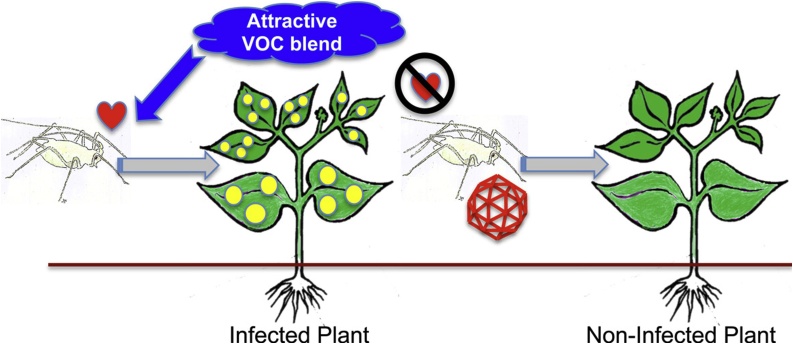
A cartoon depiction of the ‘attract and deter’ virally-induced plant phenotype. Certain non-persistently-transmitted plant viruses induce metabolic changes in infected plants that results in the emission of aphid-attracting volatile organic chemical (VOC) blends. In this scenario, an aphid may be attracted to an infected plant but brief feeding and sampling of epidermal cell contents reveals to the insect that virus infection has induced the accumulation of distasteful compounds. This deters the aphid from settling and will cause it to move on to find a more suitable host. During the sampling feed, viral inoculum will have been acquired (depicted by the icosahedron). Thus, induction of the ‘attract and deter’ virally-induced phenotype will increase the likelihood of an aphid transmitting inoculum to a non-infected plant. Based on findings and analyses by Carmo-Sousa et al. (2014), Donnelly et al. (2019); Mauck et al. (2010), and Westwood et al. (2013).

The seminal studies by Mauck et al. (2010) and later work (Carmo-Sousa et al., 2014), which investigated the interactions of aphids (*Myzus persicae* and *Aphis gossypii*) with CMV-infected cucurbit hosts, showed that CMV manipulated its own transmission in two ways. Firstly, CMV engenders the emission by the plant of increased levels of aphid-attracting volatile organic compounds (VOCs), and secondly the virus induces synthesis of anti-feedant compounds in plant tissue (antixenosis). These virus-mediated changes in plant phenotype cause aphids to be at first attracted by the VOCs to feed upon CMV-infected plants and acquire viral inoculum, only to be subsequently repelled by the taste of the antixenotic compounds (Carmo-Sousa et al., 2014; Mauck et al., 2010) (Fig. 1). CMV has similar effects on interactions between *M. persicae* and *Arabidopsis thaliana* and between aphids and tomato (Arinaitwe W., unpublished data; Bravo-Cazar A., unpublished data; Westwood et al., 2013; Wu et al., 2017). In CMV-infected *A. thaliana*, antixenosis results from increased accumulation of 4-methoxy-indol-3-yl-methylglucosinolate (4MI3M), especially within the phloem tissue, and electrical penetration graph analysis showed that ingestion of phloem sap by *M. persicae* is consequently discouraged (Westwood et al., 2013). The virally-modified host phenotypes seen in CMV-infected cucurbits and *A. thaliana*, in which plants first attract aphids and then deter them from settling (‘attract and deter’), are likely to encourage the spread of viral inoculum by aphids from infected plants to neighbouring uninfected hosts (Donnelly et al., 2019) (Fig. 1).

Mauck et al. (2012) examined the available literature on viral manipulation of host-vector interactions and found that the majority of examples of apparent viral manipulation of vector-plant interactions by non-persistently transmitted viruses are of the ‘attract-and-deter’ type. However, in some instances, infection with a non-persistently transmitted virus renders plants more susceptible to aphid infestation by making plants more nutritious and/or less resistant to aphids; a virally-modified plant phenotype called ‘retain’ by Donnelly et al. (2019). Examples of virus-induced ‘retain’ phenotypes include: potato plants infected by potato virus Y (PVY) (Boquel et al., 2011; Castle and Berger, 1993); squash plants infected with zucchini yellow mosaic virus or papaya ringspot virus (PRSV) (Blua and Perring, 1992; Blua et al., 1994; Salvaudon et al., 2013); *A. thaliana* plants infected with turnip mosaic virus (TuMV) (Casteel et al., 2014, 2015), and tobacco (*Nicotiana tabacum*) plants infected with CMV (Tungadi et al., 2019; Ziebell et al., 2011). The beneficial effects on insects can be selective and appear to reflect the interests of the virus. For example, aphid (*A. gossypii*) survival and reproduction is enhanced on PRSV-infected squash plants but there is no benefit for the whitefly species, *Bemisia tabaci*, which does not vector PRSV (Gadhave et al., 2019). In the case of CMV, this one virus appears to be able to induce contrasting virally-induced phenotypes in different hosts i.e., ‘attract and deter’ in cucurbits and *A. thaliana* and ‘retain’ in tobacco. In Section 4 we discuss the reasons why the ‘retain’ phenotype or why pleiotropy in the effects of a virus on different hosts might ultimately enhance transmission, or benefit the vector.

## 3 Viral manipulation is conditioned by specific viral gene products

Several viral genes have been identified that cause changes in the host, resulting in extended phenotypes affecting host-vector interactions and likely to enhance virus transmission. These include genes of geminiviruses (DNA viruses transmitted in the persistent manner by whitefly vectors) and their satellite DNA molecules, several of which encode factors that suppress signalling by, or responses to, the phytohormone jasmonic acid (JA) (Li et al., 2014; Lozano-Duràn et al., 2011; Luan et al., 2013; Yang et al., 2008; Zhang et al., 2012). JA regulates, among other things, resistance to insects. Thus, suppression of JA-regulated plant genes increases plant susceptibility to whitefly infestation, encourages prolonged phloem sap feeding by these vectors, and consequently increases the likelihood that they will acquire viral inoculum (reviewed in Carr et al., 2018).

Certain gene products of viruses that are transmitted non-persistently by aphids inhibit JA-regulated signalling and gene expression. Initial studies on viral suppression of JA-dependent phenomena focused on viral proteins that were suppressors of the host’s RNA silencing systems, such as the potyviral HC-Pro or the CMV 2b protein. Expression of CMV *2b* genes in transgenic *A. thaliana* or *N. benthamiana* suppressed the induction of gene expression following spraying with methyl-JA (Lewsey et al., 2010; Westwood et al., 2014). The potyviral protein HC-Pro and the P6 trans-activator protein of cauliflower mosaic virus also suppress JA-induced changes in gene expression (Love et al., 2012; Westwood et al., 2014). However, the relationship between suppression of responses to JA by some of these viral factors and their ability to modify plant-aphid interactions is not straightforward and is complicated by direct or indirect interactions with other viral gene products. For example, when the PVY HC-Pro protein was expressed constitutively from a transgene in *N. benthamiana* it suppressed JA-responsive gene expression and increased the susceptibility of these plants to infestation by *M. persicae* (Westwood et al., 2014). However, although suppression of JA-responsive gene expression occurred in PVY-infected non-transgenic *N. benthamiana* plants, aphids confined on these plants showed a significant decrease in fecundity (Westwood et al., 2014). The 126 kDa protein of tobacco mosaic virus and the p25 protein of potato virus X, both of which are viral RNA silencing suppressors, also suppressed plant transcriptional responses to methyl-JA. However, neither virus is insect-transmitted (Westwood et al., 2014). The work suggests that the ability of viruses to suppress responses to JA has evolved in many cases for reasons other than (or in addition to) the modification of host-vector interactions. The work also shows that the influence of viruses on insect–plant interactions can emerge from the combinatorial effects of multiple virally encoded factors.

Perhaps the least ambiguous viral manipulation system studied to date for a non-persistently transmitted virus is the interaction between the potyvirus TuMV, *A. thaliana* and *M. persicae*. TuMV infection increases the susceptibility of this host to aphid infestation; an apparent example of a ‘retain’ virally modified plant phenotype. The key TuMV gene product conditioning aphid susceptibility (in part by improving the plant’s nutritional properties) is the nuclear inclusion-a protein, which appears to operate via interference with ethene (ethylene)-mediated plant signalling, rather than through interference with JA-regulated signalling (Bak et al., 2019; Casteel et al., 2014, 2015). The nuclear inclusion-a protein also influences TuMV transmission more directly by relocating virus particles in cells after piercing by aphid stylets to enhance the likelihood of binding to the acrostyle receptors (Section 1) (Bak et al., 2017).

In contrast, the distinct extended phenotypes that CMV imposes on tobacco and *A. thaliana* arise from the activity of more than one viral protein. *M. persicae* confined on tobacco plants infected with CMV consume more phloem sap, show increased survival and produce more offspring than on mock-inoculated plants (Ziebell et al., 2011). However, when the aphids were confined on tobacco plants infected with the mutant CMVΔ2b, which is unable to express the 2b protein, the aphids ingested less phloem sap, reproduced poorly and exhibited increased mortality (Ziebell et al., 2011). CMV encodes five proteins including the 2b protein, which is a counter-defence factor that acts as a suppressor of RNA silencing and other resistance phenomena (Yoon et al., 2019). It appears that in tobacco, another CMV-derived factor can trigger strong antibiosis against aphids that results in decreased survival and reproduction of the insects, and which would be deleterious to virus transmission. But during an infection with wild-type CMV the 2b protein counteracts induction of antibiosis, which is induced by the 1a protein or, less likely, by the CMV RNA 1 sequence. The 1a protein is a component of the viral replicase complex but here is acting as a stimulator of resistance to aphids (Tungadi et al., 2019; Ziebell et al., 2011). The metabolite(s) responsible for the antibiosis induced by CMVΔ2b remain unknown but levels of the insecticidal alkaloid nicotine were not increased by infection with this mutant virus, suggesting that it is not the virus-inducible antibiotic factor (Ziebell et al., 2011).

In *A. thaliana*, however, the 2b protein must be prevented from inducing antibiosis. Aphids confined on transgenic *A. thaliana* plants constitutively expressing the CMV 2b protein grew and reproduced poorly and did not recover when moved to healthy, non-transgenic plants (Watt L.G, unpublished data; Westwood et al., 2013). The antibiosis-inducing effect of the 2b protein is thought to be due to its ability to bind to and inhibit the RNA slicing activity of Argonaute 1 (AGO1), a key component of both the antiviral RNA silencing and the microRNA pathways of the host. In *A. thaliana*, AGO1 negatively regulates antibiosis against aphids (Kettles et al., 2013; Westwood et al., 2013) and the interaction of AGO1 with the CMV 2b protein allows this form of insect resistance to become active (Watt, L.G., unpublished data). The 1a protein somehow inhibits the interaction of 2b with AGO1 and ongoing work is investigating if this is due to an effect of the 1a protein on AGO1, on another host factor, or on the 2b protein (Watt L.G., unpublished data; Westwood et al., 2013). However, although the 1a protein blocks induction of antibiosis, CMV still induces a mild resistance to aphids based on feeding deterrence (antixenosis). This is elicited by the CMV 2a protein (the CMV RNA-dependent RNA polymerase), which activates an immune signalling pathway (pathogen-associated molecular pattern-triggered immunity) that in *A. thaliana* results in, among other things, increased biosynthesis of the feeding deterrent compound 4MI3M in the phloem tissue (see Section 2). Specific amino acid residues in the 2a protein are responsible for antixenosis induction are currently being characterized (Rhee, S.J., unpublished data).

As observed by Mauck et al. (2010), aphids can be attracted to infected plants by insect-perceivable VOCs (semiochemicals) that ‘deceive’ the vectors into attempting to feed and settle on an unpalatable host. Similar effects (emission of attractive but deceptive semiochemicals) have been observed in *A. thaliana* (Bravo-Cazar, A., unpublished data; Wu et al., 2017), legumes (Wamonje, F.O, and Mutuku, J.M., unpublished data) and solanaceous plants (Arinaitwe W., unpublished) infected with non-persistently transmitted viruses. In CMV-infected plants, the 2b protein is the major viral factor that induces quantitative and qualitative changes in VOC blends emitted by these plants (Groen et al., 2016; Tungadi et al., 2017). The mechanism by which the 2b protein influences VOC metabolism may depend upon its ability to disrupt microRNA metabolism (Groen et al., 2016) or by its interaction with JAZ proteins (downstream factors in the JA-dependent signalling pathway) (Wu et al., 2017), or via some combination of both mechanisms.

In CMV-infected tobacco, which exhibits a ‘retain’ virally-induced phenotype (Donnelly et al., 2019; Ziebell et al., 2011), the virus infection induces a significant increase in the emission of VOCs overall and significant increases and decreases in the proportions of specific VOCs emitted by the host (Tungadi et al., 2017). However, in free-choice assays aphids (*M. persicae*) showed no indication of bias towards settling on either CMV-infected or mock-inoculated tobacco plants (Tungadi et al., 2017). This shows that although there were extensive qualitative alterations to the VOC blend as well as an increase in the amounts of specific VOCs emitted, there was no behavioural response from the aphids. Tungadi et al. (2017) suggested that by making tobacco plants better quality hosts for aphids (as shown previously by Ziebell et al., 2011) but by not making them overly attractive, the net effect will not completely inhibit movement of CMV-bearing aphids between plants and consequent virus transmission. The work also supports the observations of Webster et al. (2010), who found that the precise proportions of insect-perceivable VOCs in a blend determined whether aphids would be attracted, repelled or oblivious. Since a significant remodelling of the VOC blend occurs following CMV infection, but does not influence vector behaviour, we can speculate that this effect has evolved for some other purpose. For example, to influence other insect types such as predators, but at this point we cannot exclude the possibility that this is an incidental effect of infection.

## 4 Epidemiological modelling reveals that viral manipulation has both anticipated and unexpected consequences for virus transmission and epidemic development

**Fig. 2:**
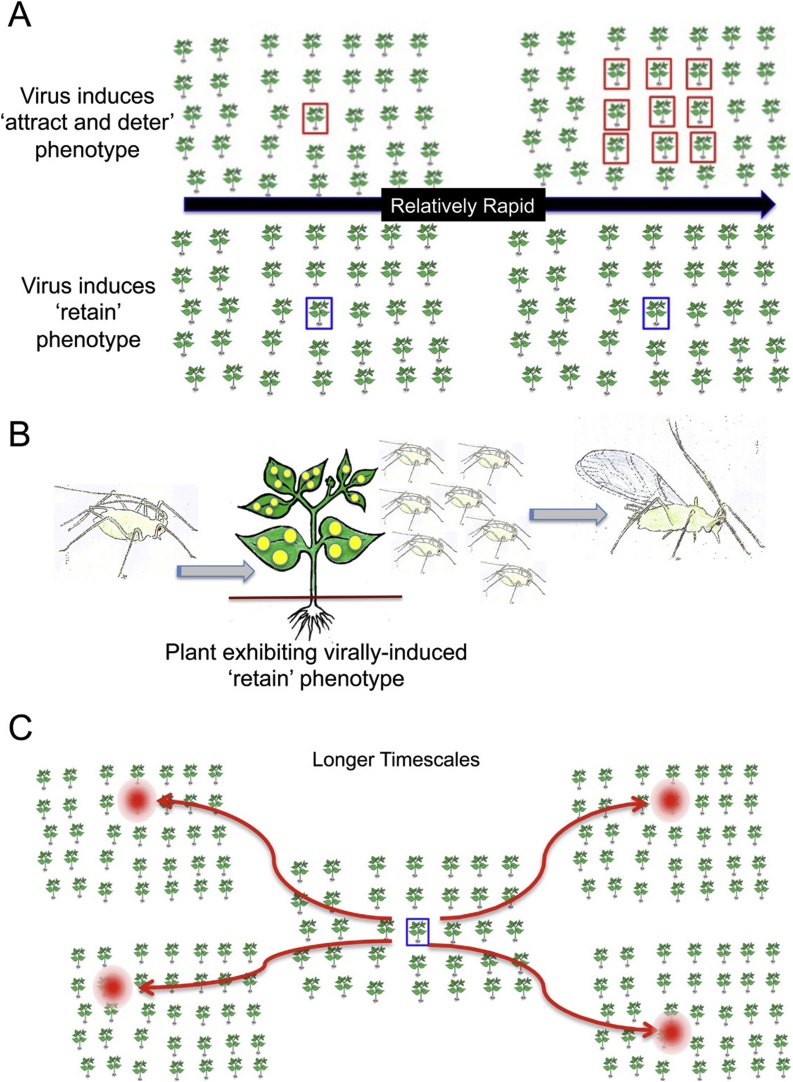
A cartoon depiction of the predicted effects of ‘attract and deter’ versus ‘retain’ virally-induced plant phenotypes on transmission of non-persistently aphid-vectored viruses. **(A)** The outputs of epidemiological modelling by Donnelly et al. (2019) support the idea that a virally-induced ‘attract and deter’ plant phenotype will engender relatively rapid local spread of virus (upper panel), whereas induction of a ‘retain’ phenotype (lower panel), will not. However, they also predict virus transmission promoted by the ‘attract and deter’ phenotype will be self-limiting since aphids will reproduce less if not settled and be exposed to increased risks of predation as they travel between plants, leading to decreased vector density. **(B)** It is predicted that a virally-induced ‘retain’ plant phenotype resulting from increased nutritional quality or decreased resistance to aphid colonisation will encourage aphids to settle, reproduce and become crowded (Donnelly et al., 2019). Crowding induces increased production of winged (alate) aphids (Braendle et al., 2006). **(C)** The modelling of Donnelly et al. (2019) predicts that enhanced production of alates on plants expressing a virally-induced ‘retain’ phenotype will result in longer-distance transmission. Although this enhancement in virus transmission may take longer to occur than that driven by the ‘attract and deter’ virally-induced plant phenotype, it was suggested that it may be more effective in launching epidemics (Donnelly et al., 2019).

Manipulation of vector behaviour by pathogens can promote epidemic development in diverse host-vector-pathogen systems (Gandon, 2018). Building on insights from previously developed modeling frameworks for non-persistent and persistent transmission of plant viruses by aphids and consequent epidemic development (McElhany et al., 1995; Jeger et al., 1998; Sisterson, 2008; Shaw et al., 2017), Donnelly et al. (2019) developed a Markov chain-based model for non-persistent transmission that incorporates viral manipulation of host-vector interactions and key aspects of aphid biology; in particular, feeding behaviour, mortality and crowding-induced production of winged (alate) aphids. Their analyses support the hypothesis that local plant-to-plant virus transmission between plants is accelerated if a virus induces the host to exhibit the ‘attract and deter’ phenotype (introduced in Section 2) (Fig. 1). A consequence of this is that while this extended phenotype increases the number of plants visited between feeds, the aphids face the disadvantage of being unable to settle long enough to grow and reproduce, as well as increased risks of not finding another suitable host upon which to alight and also increased odds of encountering predators while transiting between hosts (Donnelly et al., 2019). At this point it may also be worth noting that aphids (*M. persicae*) on CMV-infected squash plants (which exhibit the ‘attract and deter’ virally-induced phenotype) are more vulnerable to attack by parasitoid wasps (*Aphidius colemani*) (Mauck et al., 2015), which provides another disincentive to settle. This means that ‘attract and deter’ virally-induced phenotypes drive decreases in overall aphid density and ultimately to the self-limitation of spread of non-persistently transmitted viruses (Fig. 2A).

The second, and perhaps surprising, conclusion drawn from the model is that induction of a ‘retain’ phenotype can contribute to epidemic development by fostering longer-distance dissemination of viral inoculum (Donnelly et al., 2019). Those authors incorporated the effect of crowding on aphid phenology into their epidemiological model. Specifically, crowding encourages a developmental switch to an increase in emergence of alate versus non-winged (apterous) aphids (Braendle et al., 2006) (Fig. 2B). Simulation results from the model showed that if a virus fosters host susceptibility to aphid infestation, the increase in local density of apterous aphids leads to a switch from production of apterous to alate aphids that are capable of carrying inoculum over greater distances than apterous, crawling aphids (Donnelly et al., 2019) (Fig. 2C).

An additional effect flowing from the virally-induced ‘retain’ phenotype may be that vectors also benefit. It has been suggested that the changes in whitefly-host interactions induced by persistently transmitted geminiviruses provides an example of mutualism where the virus is ‘paying back’ its insect vector by providing host plants on which vector growth and fecundity are improved (Luan et al., 2013). Mutualism has also been suggested as an outcome of virus-induced susceptibility to aphids caused by non-persistently transmitted viruses (Westwood et al., 2013; Ziebell et al., 2011). Virus-infected host plants that foster improved aphid reproduction also provide refuges that allow aphids to survive adverse conditions. Interestingly, several viruses engender improved cold and drought resilience in their hosts (reviewed in Carr, 2017 and Roossinck, 2019) and this can also benefit the performance of vectors on virus-infected plants (Davis et al., 2015). It is possible to envisage virus-infected plants exhibiting two virally-induced phenotypes (increased aphid susceptibility plus improved resilience to environmental stress). These plants may then act as progenitors for subsequent generations of virus susceptible plants, as well as starting points for the spread of virus-bearing aphids to launch new epidemics.

## 5 Concluding comments

Recent years have witnessed significant advances in our understanding of how semiochemicals and other plant metabolites assist aphid vectors to locate plants and subsequently choose or reject them as hosts, and how viruses manipulate synthesis of these host cues to promote their own transmission. Additionally, we are beginning to understand at the molecular level how specific viral gene products, often working in concert, reprogram host genetic and biosynthetic networks to modify host-vector interactions. At larger scales, we are modeling the consequences of viral manipulation of host phenotypes on the development of viral epidemics.

These insights are timely. Current approaches to control of insect-vectored plant viruses in agriculture depend largely on the application of pesticides (Westwood and Stevens, 2010). However, insecticide efficacy is declining due to evolution of resistance in target insects and off-target effects of insecticides on beneficial insects are leading to restrictions on their use (Godfray et al., 2015). Meanwhile, global warming threatens to extend the geographic ranges and increase the populations of insect vectors (Canto et al., 2009). Developing new means of combating vectored transmission of plant viruses is thus a matter of urgency. A deeper understanding of how VOCs and other cues affect virus transmission by insects and how these can be manipulated or subverted (taking lessons from the viruses that have evolved to exploit plant-insect communication) could inform new methods to minimise disease spread. Similar insights into how plants and insects communicate via chemical signals have led to successful mixed cropping systems that inhibit spread of lepidopteran pests (Pickett and Khan, 2016) and we believe that similar approaches could be devised to disrupt vector-mediated virus transmission.

## Declaration of competing interests

None.
